# The Psychological and Professional Burden Experienced by Nurses Who Worked in COVID-19 Clinics during the Pandemic: A Content Analysis

**DOI:** 10.3390/clinpract13020038

**Published:** 2023-03-09

**Authors:** Areti Stavropoulou, Maria Prasianaki, Dimitris Papageorgiou, Evridiki Kaba, Evdokia Misouridou, Chrysoula Dafogianni, Georgia Gerogianni, Martha Kelesi

**Affiliations:** Department of Nursing, Faculty of Health and Care Sciences, University of West Attica, Ag. Spyridonos Str., 122 43 Athens, Greece

**Keywords:** COVID-19, nurses, nursing care, psychological burden, professional burden, nurses’ experiences, content analysis

## Abstract

Introduction: Since the beginning of the pandemic, nurses have played a key role in providing care for COVID-19 patients. Infection risk and fear, use of personal protective equipment, and social isolation were related to high levels of stress and extreme psychological drain among front-line healthcare providers. Aim: The aim of this study was to explore how front-line nurses experienced psychological and professional burdens during the coronavirus outbreak. Material and Methods: The study used a qualitative research design. Semi-structured interviews were applied as the method of data collection. Ten nurses from COVID-19 wards and units of two general public hospitals participated in the study. A content analysis approach was employed to analyze the data. Results: Data analysis revealed two main categories, namely: (A) front-line experience “From fear to empowerment”, and (B) caring and management “From powerlessness to adaptation”. Eight sub-categories were developed and included within the corresponding main categories. The study’s findings demonstrated that the pandemic caused significant psychological and professional strain on front-line nurses, with feelings of fear, anxiety, and uncertainty predominating. Nonetheless, the process of adaptation and adjustment brought about sentiments of self-actualization and empowerment. Conclusions: A thorough understanding of the psychological and professional burden experienced by the front-line nurses is crucial to ensure that nurses receive appropriate support and that quality care is sustained under highly demanding healthcare conditions.

## 1. Introduction

In January 2019, the first case of COVID-19 was officially recorded in Wuhan, China. Since then, COVID-19 has reached pandemic dimensions, with devastating effects on public health, economies, and societies. Before March 2022, about 439 million people were infected worldwide and 6 million people died from the disease [1].

Since the beginning of the pandemic, nurses have been on the front lines of caring for patients with COVID-19.

Many studies have been conducted highlighting the psychological impact of caring for COVID-19 patients on front-line healthcare professionals, including post-traumatic stress (PTSD), psychological distress, mental exhaustion, anxiety, and depression [2,3]. Other studies have demonstrated that occupational stress and burnout are closely associated to the fear and the risk of infection, the use of personal protective equipment, the social isolation, and the feelings of uncertainty that front-line healthcare professionals experienced during the pandemic era [4,5,6,7]. Emotional distress and burnout were exacerbated when there was insufficient emotional support, inadequate safety measures, or insufficient institutional support in the working environment [8].

Furthermore, the heavy workload, the need for systematic performance of specific COVID-19 diagnostic tests such as PCR, and the care of family members were mentioned as factors that increased the risk of developing mental health problems among front-line health professionals [9]. In addition, prior mental illness, underlying organic disease, family problems, and lack of personal protective equipment negatively affected nursing staff working with COVID-19 patients [10,11]. Specifically, front-line nurses were more prone to symptoms of anxiety and insomnia as well as higher levels of stress and subjective psychological burden than other healthcare professionals [12,13].

Other studies referred to barriers faced by nurses during the care of patients with COVID-19, such as limited information about COVID-19 and a perceived lack of support by upper management [14].

Research on the COVID-19 pandemic in Greece has mostly focused on examining the socioeconomic effects and the health system’s response to the healthcare crisis [15,16,17]. Furthermore, the psychological and mental health problems experienced by healthcare providers and the general public during the coronavirus outbreak were explored [18,19,20]. Similar issues were discussed in the international literature, suggesting that psychological intervention strategies and measures taken at a governmental level may prevent feelings of uncertainty and anxiety, promote feelings of security, and improve health professionals’ mental health during epidemics [21]. 

Most of the research studies conducted since the beginning of the pandemic have relied on quantitative methodological approaches in order to analyze the psychological impact of healthcare professionals working on the front lines. However, few of these appeared to focus on health professionals and nurses’ personal views and experiences [22,23]. 

The aim of the present study was to explore how nurses who worked in COVID-19 units and wards experienced the psychological and professional burden caused by the pandemic. In-depth investigation of nurses’ views and experiences while caring for COVID-19 patients may contribute to the existing knowledge by providing a thorough understanding of nurses’ psychological and professional burdens caused by the coronavirus outbreak. 

## 2. Materials and Methods

### 2.1. Design

A qualitative research approach was used based on the principles of content analysis. This is considered the most appropriate method for investigating human experiences, emotions, attitudes, and opinions [24]. It is a method that makes the world more visible by studying the unique experience of the individuals in depth within their natural and complex environments [24,25]. While the main goal of research is to inform science and to develop new knowledge through description, prediction, and generalization, qualitative approaches enable the researcher to make sense of the existing reality and to describe and explain the social world by focusing on the unique nature of the phenomenon under study [26,27]. 

### 2.2. Context and Participants

Nurses who have worked in COVID-19 wards and units of two general hospitals (Site H1 and Site H2) for at least 6 months constituted the study population. These criteria were considered essential for the participants to have sufficient experience in caring for COVID-19 patients and, thus, to be able to provide rich information about the topic under investigation. 

A purposeful sampling strategy was used to recruit the study participants. One member of the research team contacted the hospital nurse directors and nurse managers of COVID-19 wards and units. Detailed information was provided regarding the nature and purpose of the research for gaining nursing services’ trust and support during the recruitment phase. The study was advertised within the hospitals through personal contact with the nursing personnel. Meetings with individual nurses who were interested in discussing issues concerning the study were organized, and all appropriate explanations were provided. Finally, ten nurses from the two study sites agreed to participate. They were all involved in care provided in COVID-19 wards and units. Their overall working experience ranged from 2 to 31 years, and they were all university graduates. None of the study participants withdrew before the completion of the research. Demographic characteristics of the study sample are presented in Table 1. 

### 2.3. Data Collection

Data collection took place in H1 and H2 from March to August 2022. Semi-structured interviews were used for data collection. This type of interview enables the researcher to maintain some control over the content of the interview, but at the same time, the participants have the opportunity to express themselves freely and to thoroughly reveal their views and experiences [28]. The interviews were carried out by a member of the research team who was a nurse trained in qualitative research methodology and in developing appropriate interviewing skills. The location and the time of the interviews were chosen by the participants. All interviews were conducted within the hospitals, in a private office, taking all protective measures against COVID-19. The location was carefully selected to protect the confidentiality of the discussion and to ensure that any interruptions during the interview process would be avoided. A tape recorder was used for data collection purposes; thus, permission was granted by each participant before conducting the interview. 

Τhe interview scheme included an introductory open-ended question asking the participants to describe their experience of working in a COVID-19 ward or unit during the pandemic. Prompts and further questions were used whenever necessary, with regard to the feelings of the participants, the psychological problems, the burdens, and the challenges faced during the pandemic. Finally, the participants were invited to share an incident or an experience that scarred them at work during the pandemic.

All interviews were conducted in the Greek language. They lasted from 20 to 35 min, ending when the participant had nothing more to add to his/her narrative. Transcription of each interview took place immediately after its completion. To assure the accuracy of the translation and the precise depiction of the participants’ quotations the backward translation technique was applied. 

Data saturation in the present study was achieved, as after the completion of the eight interviews, the information provided by the participants was repeated and no new data emerged that would allow the researcher for further coding. 

### 2.4. Data Analysis

Inductive content analysis method was used to analyze the content of the interview. According to Elo and Kyngas [29], the process of content analysis includes three main phases: preparation, organization, and reporting of the results. In the preparation stage, the researcher collected and gained insight of the data. The text was read repeatedly and words and phrases were selected to be the units of analysis. In the organization phase, abstraction, open coding, and formulation of categories took place. Next to each paragraph, keywords or phrases representative of the content were highlighted. Then, words and phrases involving common concepts were coded and categorized. The formed categories were representative of the concepts emerging from the data. In the reporting phase, the results were presented, with the content of each category/subcategory illustrating the topic under investigation. 

### 2.5. Ethics

Before the commencement of the study, a research protocol was submitted and approved by the Scientific Committee of the two study sites (H1 and H2). Approvals were granted with reference numbers 1897-08/02/2022 and 2325-22/06/2022, respectively. The purpose and the scope of the research, the data collection, and the data management methods were precisely explained to the participants by the researcher. Particular emphasis was placed on their voluntary participation in the research, and written permission was sought from each participant for the use of a tape recorder before the interview process began. Participants were encouraged to openly discuss their experiences working in the COVID-19 wards or units. It was also made clear to them that they might withdraw from the research at any moment if they wished, without any penalty. 

Anonymity and data confidentiality were ensured before, during, and after the interview by using a code number for each participant. The nurses were informed that the interview data would be used exclusively by the researcher for scientific purposes only. It was also made clear that the data would be stored as an encrypted document on the researcher’s (MP) personal computer and destroyed after a reasonable period of time. Access to the data would likely be for reasons of checking and ensuring the reliability of the results. Only authorized members of the research team would be able to have access to and readability of the data folder. Participants were also informed that they could have full access to the results of the study if they wished. A consent form for the study was signed by all participants before their involvement in the research process. 

### 2.6. Credibility of Research

The researchers used various strategies in the present study to ensure the trustworthiness and the rigor of the research design and the method that was followed [27]. Analyst triangulation was applied, as two experienced researchers were engaged in the analysis and interpretation of the data, for the purpose of ensuring the credibility of the study findings. The researcher used also a research diary to examine her own values and assumptions and to assess how these may have influenced decision making throughout the research process [30]. 

### 2.7. Findings

Ten nurses, graduates of higher educational institutions (HEIs) and aged between thirty-three and fifty-five years, participated in the present study. All participants had work experience in COVID-19 wards and units for at least six months. Nurses in COVID-19 units provided care to patients who were in critical condition, while patients in stable condition were treated in COVID-19 wards. The demographic data of the participants are presented in Table 1.

Two main categories emerged from the data analysis, which were named: (A) front-line experience “From fear to empowerment”; and (B) caring and management “From powerlessness to adaptation”. A total of eight subcategories were formed and accordingly included in each category. The main categories and subcategories are presented in Table 2.

#### 2.7.1. A. Front-Line Experience “From Fear to Empowerment” 

Front-line nurses who worked in COVID-19 sectors came across unique experiences during the pandemic’s outbreak. Psychological drain and feelings of fear, anxiety, uncertainty, and grief were reported, while gradual adaptation and transition to the new situation led to feelings of empowerment, self-awareness, satisfaction, and relief.

##### A1. Living in Fear 

According to the participants’ statements, emotions such as anxiety and fear appeared to prevail amid the COVID-19 outbreak. These emotions arose from the unprecedented health crisis, the risk of infection, and the nurses’ lack of knowledge and experience in managing similar situations.

*“The truth is that it was something unprecedented for me, I was very stressed...stressed because I didn’t know this job…suddenly I had to deal with something very difficult, without having the proper experience and training… I was afraid… of being infected and transmit the virus to my family...the whole experience in the COVID Unit was very difficult… I was going back home crying...”*

N1

*“I was very anxious, I didn’t know what COVID 19 is… It was a stressful time, that’s what has been left from my front-line experience during the pandemic, the fear of the unknown….”*

N8

*“It was it a creepy situation...I was scared, I didn’t know how to handle my stress.”*

N10

The transmission of the virus to fellow nurses, along with the information conveyed by the mass media about COVID-19, magnified the feelings of fear and anxiety.

*“We didn’t know what COVID was. The way the information was conveyed through the mass media caused terror. We were anxious how well we would do what we were assigned to… there were so many obstacles… We experienced also a psychological shock when we saw our colleagues to get sick from the virus…”*

N7

##### A2. Coping with Grief

The study participants recalled the painful situations that caused sadness and emotional drain, mainly due to patients’ deaths.

*“We had a lot of deaths, …, I don’t even want to think about it...it was dreadful, I was affected so much, I was so emotionally strained … so many deaths…for many months we had only one discharge, everyone was dying...”*

N8

The difficulties arising from the severity of the disease and the poor prognosis of the patients created negative emotions, as well as psychological and physical constraints.

*“It is a difficult experience, it wears you out both physically and psychologically, because it is not only the physical fatigue... we had to intubate young people, mothers and fathers with the family outside waiting for information, it was difficult....; we had to cope and survive...”*

N4

Positive thinking was referred to as an ally for the patients; however, the nurses had to cope on a daily basis with frustration and grief.

*“Patients who believed in themselves did it better...those who didn’t try…died...just like that! The high fever, the feeling of suffocation, the shortness of breath, these dreadful sounds …. the sound of the inhalers, the sound of the oxygen supply were daily issues that the patients had to face...and we had to cope with.”*

N7

##### A3. Experiencing Empowerment 

Along with the aforementioned difficulties, the participants reported positive emotions, new knowledge and skills, strength, and self-confidence.

*“Well, I was anxious and scared, but I also had positive feelings at the same time… I was learning something new I was gaining more qualifications and strength...my work in the COVID Unit, is an excellent experience, it broadens my horizons it gives me confidence…I finally came out of this stronger as a person and as a nurse....”*

N1

*“I learned to deal with difficulties, very quickly I gained knowledge and experience… I became stronger.”*

Ν9

Feelings of empowerment helped nurses to improve care, eliminate stress, communicate better with the patients, and appreciate life’s values.

*“...it is positive experience for me, I learned things, I realized the life values, …through this adventure I gained things, I learned how to protect myself better, how to protect my colleagues, how to take better care of my patient and I appreciated what it’s like not to have all that... it was very positive for me.”*

N2

Social support also appeared to empower nurses and help them continue their difficult work.

*“The people embraced us, gave us the courage to move on.... they treated us as heroes.... they gave us strength to continue...”*

N3

##### A4. Obtaining Satisfaction and Relief

Having experienced intense and painful emotions, nurses were able to spot certain moments of relief and joy, especially when the patients’ health was improving and they were finally discharged.

*“There was one patient that we all remember… he was around 50s and he had really a nasty experience of COVID-19…psychologically and physically. He was seeing all the patients around him who were around his age, intubated and then dying…This patient admitted in September 2020 and discharged at Christmas Eve 2020 standing up! He believed in himself, he believed in his power, he trusted us ...he left applauding, the whole team gathered, it was a victory…his wife with his children were waiting across the street and we remember it…it was such a relief… it was Christmas... he brought to us chocolates to say thank you!”*

N7

Feelings of self-actualization, satisfaction and improved morale and self-esteem were also reported by the front-line nurses. Being at the front line, providing good care to COVID-19 patients, and having a successful therapeutic outcome increased the nurses’ satisfaction with their self-confidence and professional commitment.

*“I contribute to humanity… you are at the front-line when others stayed at home! You are at the front ...this is a great challenge!”*

N10

*“I felt that I offered a lot, I am satisfied with this... there is nothing else, not financial benefits, nothing ... I am satisfied simply because the patient is here, alive and satisfied... There were young people for whom we wished not to be intubated, we were fighting for that...; and they managed to avoid intubation... we were happy about it and that was a great satisfaction, … those who were not intubated, who were saved are still come and thank us… the most pleasant thing was to see the patients to recover and go home. This was very important for me…the greatest satisfaction…”*

N5

#### 2.7.2. B. Caring and Management “From Powerlessness to Adaptation”

Nurses were forced to transform their workplaces and to adapt new guidelines and new ways of managing patients rapidly and effectively. Care was confronted with several barriers; however, nurses found ways to overcome the obstacles and adapt to the new conditions successfully.

##### B1. Encountering Problems

Appropriate staffing and a lack of experienced personnel were reported as the most significant problems that nurses faced during the pandemic.

*“It would be much easier if there were more nurses in the Unit to help, ... some things would be much easier....”*

N1

*“We organised a COVID-19 ward from scratch, at the beginning everything was tough…. the nursing personnel was newly qualified, just 5 or 6 nurses were experienced and these people had to deal with this difficult situation... we didn’t have assisting personnel and we had patients in critical condition... they couldn’t even hold a glass with water…we did everything for them, it’s tiring, our work is tiring....”*

N4

In addition, exhaustion and communication problems between nurses and patients due to PPE were reflected.

*“Suddenly we had to work without proper training, using PPE, wearing glasses ad masks.... we faced problems with the uniform, it’s not easy... the effort is twice as much...”*

N7

*“It was difficult, you get very tired with the PPE, you can’t listen, you can’t speak, you have to shout...we had communication problems we had problems to be heard, to be understood...”*

N5

##### B2. Living in Isolation

To a further extent, front-line nurses during the pandemic experienced social, professional, and personal isolation due to the possibility of spreading the virus to other co-workers, family, and friends.

*“...Oh, yes.... I was tired of all this.... we were treated by other hospital colleagues (those who were not working with COVID patients) as lepers...this bothered me,....I remember the first days (of the pandemic) we went to the haematology department and when we entered, some colleagues were so scared, that they told us to stand at the door and wait outside, we could see a terror in their eyes, ...it was the terror of the unknown ...”*

N2

*“We received a kind of ‘racism’ from our own colleagues, as if we were those working with COVID patients ...there were also people who told us that we brought the virus to the hospital.”*

N7

Similar phenomena were reported in relation to family and social activities, resulting in some cases in self-isolation.

*“Τhey (the friends) were looking at me with doubt because I was working with COVID patients... I told them that I was more protected than they were, it was more likely that I would be infected by them, not the other way around...they could not understand it.”*

N9

*“We isolated ourselves from family and friends …, we didn’t even go out for a coffee, nothing.... we kept the safety measures very strictly and yet we remained isolated...you leave the work very stressed and you don’t have a way out...not even to meet a friend… to go out for a walk.…”*

N6

##### B3. Going Overboard

Nurses reported that flexibility and altruism were needed to overcome the various problems related to organization and staffing. Personal effort and teamwork were employed to confront these difficulties.

*“We had very short time to organise things but we did it, each of us with his own personal effort but also all together... and I feel that this situation has brought us together as colleagues, we are more attached now ....”*

N8

*“It was wartime conditions, we were leaving the unit for a while just to pop in and help our colleagues in the ward, it was not our duty but we did it.”*

N9

*“I made friends during this war, not only colleagues... There were many problems, … we managed everything without complaints… there were difficulties but also pleasant moments …and being with new colleagues, young people was also a pleasant note…”*

N5

Communication and patient management were supported by the nurses with spontaneity and ingenuity.

*“We put in our uniforms cute decorations, Christmas trees and stars, we had our names on them… and even the patients who were obviously stressed by their condition, by hearing our voice and seeing our uniform, they were encouraged… and I could see it in their eyes a sense of safety and a joy and it was wonderful, … wonderful....”*

N2

##### B4. Getting Growth Opportunities

Through a demanding healthcare environment consisting of constant changes, the nurses appeared to recognize some opportunities for growth and transformation. For some nurses, the pandemic was an opportunity for professional development. Interdisciplinary collaboration was enhanced, and organizational issues were revisited.

*“I felt that my working conditions were improved. It is the first time in my life that I have a permanent contract as a nurse.... It is very important ... we had very good cooperation with the doctors, ...we had solidarity... ”*

N4

*“We organised our work more intensively… we may have been working hard but we were more focused… with no interferences…COVID came and taught us things that we can now appreciate.”*

N7

At the personal level, growth opportunities were reported in terms of self-awareness and introspection.

*“It was a time when everything was stopped... this helped us to move forward, we found time for ourselves, we revised our priorities, our relationships, we realised that nothing is for granted... and we gave priority to our health...”*

N8

*“At the beginning we faced huge difficulties, after a while everything was better, we learned how to manage the work, the problems, ourselves, we found ways to cope…In other words, we slowly learned to manage things, we learned how to deal with the problems to our advantage.”*

N2

## 3. Discussion

The present study demonstrated that nurses experienced painful experiences during the pandemic which affected them both physically and psychologically. The nurses’ psychological constraints were mainly associated with feelings of fear, uncertainty, anxiety, isolation, social restrictions, exhaustion, and powerlessness. However, through the unfavorable working and social conditions, they managed to discover ways to cope and adapt to the new situation, responding positively at both a personal and professional level.

### 3.1. Front-Line Experience “From Fear to Empowerment”

The results of this study demonstrate that fear and anxiety were the dominant emotions experienced by nurses at the beginning of the pandemic, as these were related to anxiety and fear of the unknown as well as the risk of infection and death. Similar results have been reported in the literature, since health professionals’ fear of the virus was characterized as “virus shock” [31], while high levels of fear and anxiety were found in front-line nurses due to the unfamiliar professional environment and the adverse working conditions [32]. Moreover, fear of family contamination appeared to be a major concern of nurses globally, causing high levels of stress and anxiety [33,34].

This study also found that unclear information resulted in psychological restraints and negative feelings. The relevant literature emphasizes that the uncertainty, which was caused by a lack of knowledge, appropriate information, and unpredictable changes triggered by COVID-19, may have led to nurses’ emotional distress and psychological drain [35,36].

Nurses experienced deaths of their patients, relatives, and colleagues. The findings of the present study indicate the grief and frustration that the nurses experienced due to the high mortality rates during the pandemic. In agreement with our findings, several studies focused on nurses’ emotional discomfort caused by dying patients, communication barriers, the difficulty of breaking bad news to families, and the stressful working conditions were due to the severity of the disease and the poor prognosis of the patients [37,38]. The isolation ward was described as a depressing, high-risk, and stressful place, causing grief and frustration [39,40].

Similarly, to the findings of the present study, relevant data highlighted that despite the negative emotions, nurses acquired professional growth, adaptability skills, and enhanced psychology [41]. Liu et al. [42] underlined that the professional dedication of nurses was the most important motive for overcoming the difficulties caused by the COVID-19 pandemic. Furthermore, a supportive environment, caring leadership, and patients’ well-being were referred to as empowering and fulfilling factors for nurses, as they uphold quality nursing care, professional commitment, and job satisfaction in times of healthcare crises [2,39].

### 3.2. Caring and Management “From Powerlessness to Adaptation”

Understaffing, lack of experienced personnel, novel working conditions and guidelines, engagement with non-nursing duties, use of PPE, and communication barriers were perceived as major organizational problems in COVID-19 wards and units. In the same vein, relevant data demonstrate the problem of miscommunication and the lack of physical contact with patients due to PPE [43,44].

The unexpected changes in the health system due to the pandemic negatively affected nurses’ job performance, while the rapidly shifting environment necessitated organizational, professional, and personal changes, which led to dissatisfaction and stress [45].

According to our findings, a variety of challenges experienced by nurses involved isolation from colleagues and friends due to the fear of transmission. More specifically, isolation at a professional level has been reported as a factor leading to psychological strain for nurses [34]. Similar studies have referred to exclusion phenomena during epidemics, which could be eliminated through administrative and psychosocial interventions such as training, stress management programs, counseling, and financial support [46,47,48].

Flexibility and professional commitment stimulated nurses to adapt to the new organizational conditions during the pandemic era. Similar studies stated that nurses overcame the organizational barriers by remaining committed to their roles and their professional values. Ethical and professional commitment and humanitarian concern for the patients were mentioned as factors that helped nurses to overcome problems during the pandemic and adapt to the new working conditions [49]. This adaptation required time and personal sacrifices, such as reductions in nurses’ rest time and increased time spent exposed to the virus [49,50].

The relevant literature states that nurses not only adapted to the new demanding working conditions during the pandemic, but also improved their experiences by developing professional responsibility and self-reflection [38]. Similarly, the findings of the present study highlight the professional, interpersonal, and social growth that our participants experienced during the COVID-19 outbreak.

## 4. Limitations of the Study

Nurses who worked in COVID-19 wards and units at two public general hospitals in an urban region comprised the study sample. The nurses’ heavy workload during the time that the research was conducted, as well as their strict working schedule, may have limited their enthusiasm and ability to participate in the study. A future study with a sample from different health care organizations and from a wider geographical area is suggested in order to enrich the scientific knowledge of the topic under investigation. The results of this study should be considered under these constraints.

## 5. Conclusions

The findings of the present study showed that anxiety, fear, and uncertainty were the dominant emotions experienced by nurses working in COVID-19 wards and units. The sudden changes caused by the pandemic negatively affected nurses and patients’ care. However, nurses identified opportunities for professional and personal development, strengthened themselves professionally and scientifically, and enhanced their roles through interdisciplinary collaboration with other health professionals. The findings of this study provide important information for healthcare professionals regarding the psychological and professional burden experienced by nurses who worked in COVID-19 wards and units during the pandemic. Further research is recommended focusing on nurse managers’ and healthcare policy makers’ experiences and concerns amid the coronavirus outbreak.

## Figures and Tables

**Table 1:** Participants’ demographics.

Participant Code	Age	Gender	Marital Status	Education	Years of Employment (Total)	Working Position	Hospital	Duration of Employment in COVID-19 Ward/Unit (in Months)
N1	38	F	Single	University/MSc	15	Staff Nurse	H 1	ICU COVID–11 months
N2	43	F	Married	University/MSc	2	Staff Nurse	H 1	ICU COVID–11 months
N3	46	F	Married	University	20	Staff Nurse	H 1	COVID ward–6 months
N4	33	F	Married	University	10	Staff Nurse	H 1	COVID ward–24 months
N5	47	F	Married	University/MSc	24	Staff Nurse	H 2	COVID ward–16 months
N6	34	F	Single	University/MSc	13	Staff Nurse	H 2	COVID ward–16 months
N7	55	F	Married	University	31	Nurse Manager	H 2	COVID ward–24 months
N8	39	F	Married	University/MSc	12	Staff Nurse	H 2	ICU COVID–8 months
N9	28	M	Single	University	2	Staff Nurse	H 2	ICU COVID–12 months
N10	29	M	Single	University/MSc	8	Staff Nurse	H 1	ICU COVID–12 months

**Table 2:** Main categories and subcategories that emerged from the analysis of findings.

Main Categories	A. Front-Line Experience “From Fear to Empowerment”	B. Caring and Management “From Powerlessness to Adaptation”
Subcategories	A1. Living in fear A2. Coping with grief A3. Experiencing empowerment A4. Obtaining satisfaction and relief	B1. Encountering problems B2. Living in isolation B3. Going overboard B4. Having growth opportunities

## Data Availability

Data generated during the present study are not able to be shared due to issues of subjects’ privacy and confidentiality.
